# 3D Architecture of the *Trypanosoma brucei* Flagella Connector, a Mobile Transmembrane Junction

**DOI:** 10.1371/journal.pntd.0004312

**Published:** 2016-01-28

**Authors:** Johanna L. Höög, Sylvain Lacomble, Cedric Bouchet-Marquis, Laura Briggs, Kristin Park, Andreas Hoenger, Keith Gull

**Affiliations:** 1 Sir William Dunn School of Pathology, University of Oxford, Oxford, United Kingdom; 2 The Boulder Laboratory for 3D Electron Microscopy of Cells, Department of MCD Biology, University of Colorado, Boulder, Colorado, United States of America; 3 FEI Company, Hillsboro, Oregon, United States of America; McGill university, CANADA

## Abstract

**Background:**

Cellular junctions are crucial for the formation of multicellular organisms, where they anchor cells to each other and/or supportive tissue and enable cell-to-cell communication. Some unicellular organisms, such as the parasitic protist *Trypanosoma brucei*, also have complex cellular junctions. The flagella connector (FC) is a three-layered transmembrane junction that moves with the growing tip of a new flagellum and attaches it to the side of the old flagellum. The FC moves via an unknown molecular mechanism, independent of new flagellum growth. Here we describe the detailed 3D architecture of the FC suggesting explanations for how it functions and its mechanism of motility.

**Methodology/Principal Findings:**

We have used a combination of electron tomography and cryo-electron tomography to reveal the 3D architecture of the FC. Cryo-electron tomography revealed layers of repetitive filamentous electron densities between the two flagella in the interstitial zone. Though the FC does not change in length and width during the growth of the new flagellum, the interstitial zone thickness decreases as the FC matures. This investigation also shows interactions between the FC layers and the axonemes of the new and old flagellum, sufficiently strong to displace the axoneme in the old flagellum. We describe a novel filament, the flagella connector fibre, found between the FC and the axoneme in the old flagellum.

**Conclusions/Significance:**

The FC is similar to other cellular junctions in that filamentous proteins bridge the extracellular space and are anchored to underlying cytoskeletal structures; however, it is built between different portions of the same cell and is unique because of its intrinsic motility. The detailed description of its structure will be an important tool to use in attributing structure / function relationships as its molecular components are discovered in the future. The FC is involved in the inheritance of cell shape, which is important for the life cycle of this human parasite.

## Author Summary

*Trypanosoma brucei* is an uni-cellular parasite transmitted to humans and cattle by the bloodsucking tsetse fly. Once swimming in the mammalian bloodstream, it causes the devastating African sleeping sickness in humans and nagana in cattle. During its complex life cycle, it undergoes many cell shape changes, which are important for efficient parasite transmission. Here, we have studied a cell structure intrinsically involved in shape acquisition during division of the *T*. *brucei* life cycle form that multiplies in the fly midgut. Using electron tomography we show the 3D architecture of a motile cellular junction that slides with the tip of the growing new flagellum along the side of the old flagellum. This enables the new flagellum to zip in to the cell body structure alongside the old flagellum after which the cleavage furrow is established between these two flagella, producing two daughter cells of similar cell shape. We present here a detailed architectural overview of this junction; we show that it matures with time and pushes the old flagellum’s axoneme sideways as it passes. This structural map enables insight into the function of this extraordinary mobile cellular junction.

## Introduction

Cellular junctions are crucial for the formation of tissues, pathogen/host cell interactions and communication between cells, e.g., the plasmodesmata in plants and the gap junctions in animals. However, junctions can also exist within a single cell, such as the top connectors between sterocilia and the kinocilium on outer hair cells in the ear [1].

*Trypanosoma brucei* are unicellular protozoa able to form multiple kinds of cellular junctions. These parasites cause the devastating African sleeping sickness that is transmitted to humans and cattle by the bite of an infected tsetse fly (*Glossina spp*). The ability to adapt to a changing environment is essential to their complex life cycle [2]. One such adaptation is the asymmetric intercellular junctions between the *T*. *brucei* flagellum and the microvilli in the tsetse fly salivary gland epithelia [3]. When the parasites are attached like this, the cells divide asymmetrically to generate daughter cells of a different shape. Similar cellular junctions between the flagellum and the host species tissue are also found in *T*. *congolense*, *T*. *vivax* and *Leishmania mexicana* [4–6], providing not only a physical tethering to the substrate but also a signaling opportunity such the one described between the parasitophorous vacuole and the amastigote *L*. *mexicana* flagellum [5].

Procyclic *T*. *brucei*, the form that infects the fly mid-gut, possess a single flagellum that originates in the flagellar pocket and exits the cell body near the posterior end of the cell [7,8]. The extracellular part of the flagellum contains an extra-axonemal structure called the paraflagellar rod (PFR; [9–12]), and is attached to the plasma membrane through a region called the flagellar attachment zone (FAZ; [2,3,13]. Inside the FAZ, a specific complex junctional component, the recently discovered ‘staple’ is found [12]. These are extracellular plate-like structures with fibrous connections into both the flagellum and cell body. These are, in contrast to the first example of *T*. *brucei* cellular junctions, intracellular connections, connecting one part of the cell to another.

**Fig 1:**
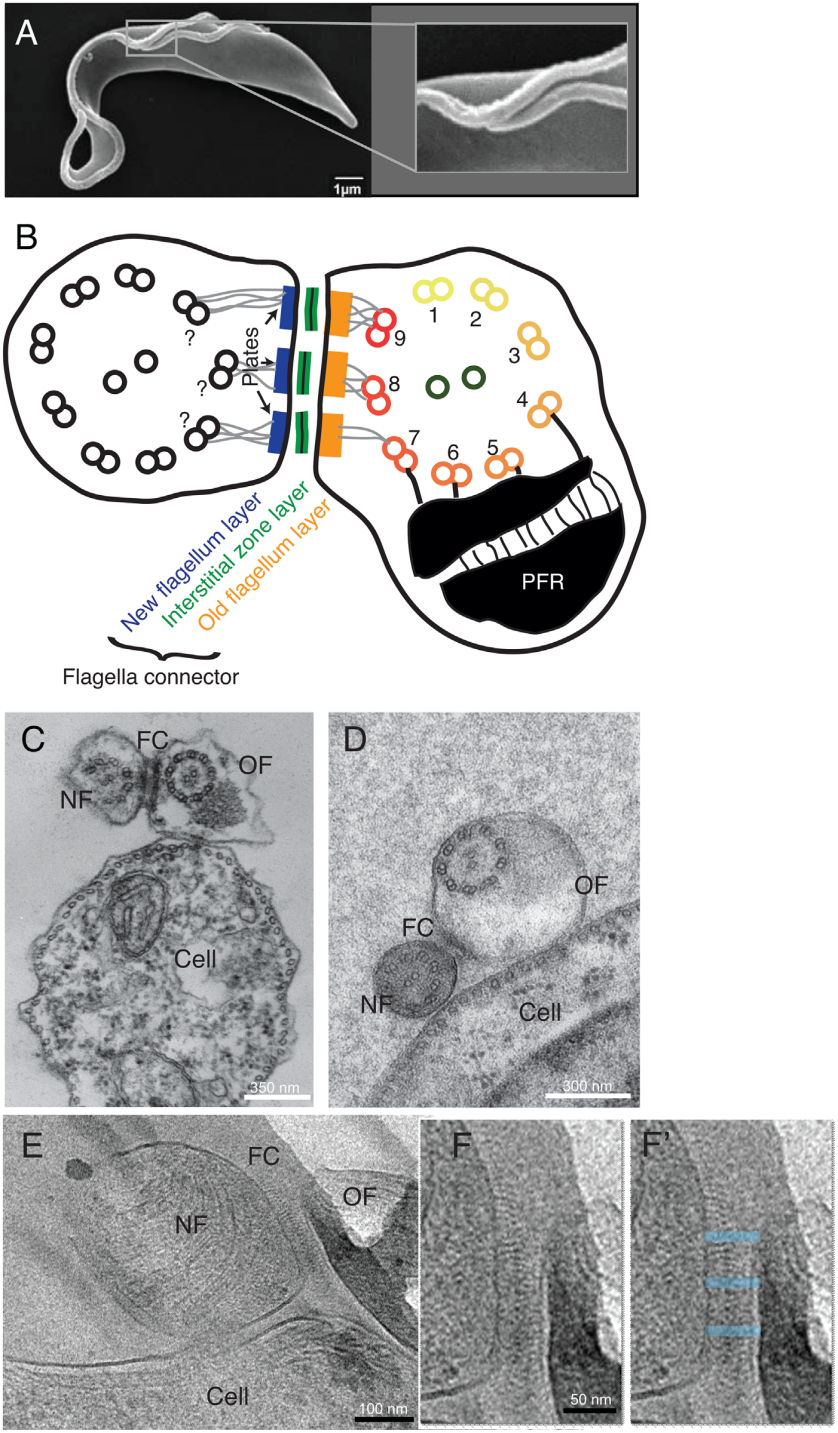
The flagella connector (FC) is a motile cellular junction spanning the membranes of both the old and new flagellum. A) Scanning EM micrograph of a procyclic T. brucei in which the new flagellum has exited the flagellar pocket and is attached to the old flagellum at the FC (see higher magnification image of this area). B) A cartoon adapted from [17] showing the known structure of the FC, as if seen in cross-section. C) A chemically fixed FC in cross section, the FC appears as electron dense layers in the new flagellum (NF) and old flagellum (OF), as well as in an interstitial zone. D) In high pressure frozen cells the FC is less apparent, the new flagellum is more electron dense and the flagella are round in profile. E) Cryo-EM of frozen hydrated sections of a FC shows a repetitive structure in the interstitial zone. F-F’) A zoomed in image of the structure in E. In F’, breaks in the repetitive interstitial densities are highlighted (blue transparent boxes).

A third cellular junction in *T*. *brucei* is the flagella connector (FC); a specialization that is unique to procyclic cells in division that are assembling a second flagellum that will be inherited by a daughter cell [5,14–16]. The FC is a mobile trans-membrane junction that links the tip of the new flagellum to the side of the old flagellum (Fig 1A; [14,17]). Once the new flagellum tip, and the FC, has reached a point roughly 50% along the length of the old flagellum, it stops migrating. From then on the tip of the new flagellum is immobile on the surface of the old flagellum, and continued flagellar growth is temporally accompanied by independently separating basal bodies and kinetoplasts [18]. The physical connection between old and new flagellum probably ensures that the elongating new flagellum copies the left-handed helical path of the old flagellum [19], facilitates flagellar attachment zone formation and thus imposes a similar cell shape on the ensuing daughter cells after division.

The basic outline of the FC structure has been described using conventional thin-section electron microscopy of chemically fixed material [14,17]. This work showed that the FC consists of a tri-laminar structure composed of three distinct electron dense layers found in the new flagellum, the interstitial space, and the old flagellum. Each layer is subdivided into three plates. Interconnecting these layers with the axonemal microtubule doublets are thin intra-flagellar filaments (Fig 1B; [7,8,17]).

However, many aspects of the FC structure and behaviour have remained elusive. For example it is not known how it moves along the old flagellum, although we do know this motion is separate from the extension of the new flagellar axoneme [9–12,18], indicating the presence of some sort of molecular motor. We have now performed (cryo) electron microscopy and (cryo) electron tomography with the hope of further clarifying the function and mechanism of this junction and its motility. The combination of techniques used has resulted in our developing a comprehensive 3D architecture, presented here, that provides insight into the physical properties of the FC.

## Results

### 2D ultrastructure of the flagella connector

To investigate the FC ultra-structure, we performed transmission electron microscopy on both formaldehyde fixed cells and cells cryoimmobilised by high pressure freezing. In thin cross-sections of chemically fixed flagella, both the new and old flagella had irregular outlines (Fig 1C). The tip of the new flagellum lay quite distant from the plasma membrane of the cell, and some of its doublet microtubules were missing[15]. The FC displayed partitions in the electron dense material (previously named “plates” [17]) throughout the interstitial zone. In high pressure frozen cells, on the other hand (Fig 1D), interstitial zone material was visible, but no clear partitions into plates were visible. In this preparation, both flagella were round in cross-section. New and old flagella are both in close proximity to the cellular surface. The new flagellum in Fig 1D had a central pair that was parallel with the underlying sub-pellicular microtubule array, but the old flagellum’s central pair axis was rotated in comparison to the cellular microtubules (Fig 1D and S1 Fig).

The FC was then examined using cryo-electron microscopy of sections cut from high pressure frozen cells embedded in vitreous ice. Because this sample preparation does not involve dehydration of the cells, nor coating of proteins with heavy metals, it displays cell structure in a close-to-native state [12,20]. This image shows the two flagella, both close to the cell membrane and between them we find the FC (Fig 1E). Distinct, regular filamentous densities project from both flagella membranes in the FC and a region of darker electron density is found in the middle of the interstitial zone. This filamentous arrangement is interrupted in three areas by smoother electron densities across the FC (Fig 1F–1F’; blue boxes).

We conclude that studying the FC ultra-structure using various sample preparations and imaging techniques yields new information about its ultrastructure. Therefore, we progressed to study its 3D structure using electron tomography of the conventional chemically fixed, high pressure frozen and vitrified samples.

### 3D architecture of the FC visualised by electron tomography

**Fig 2:**
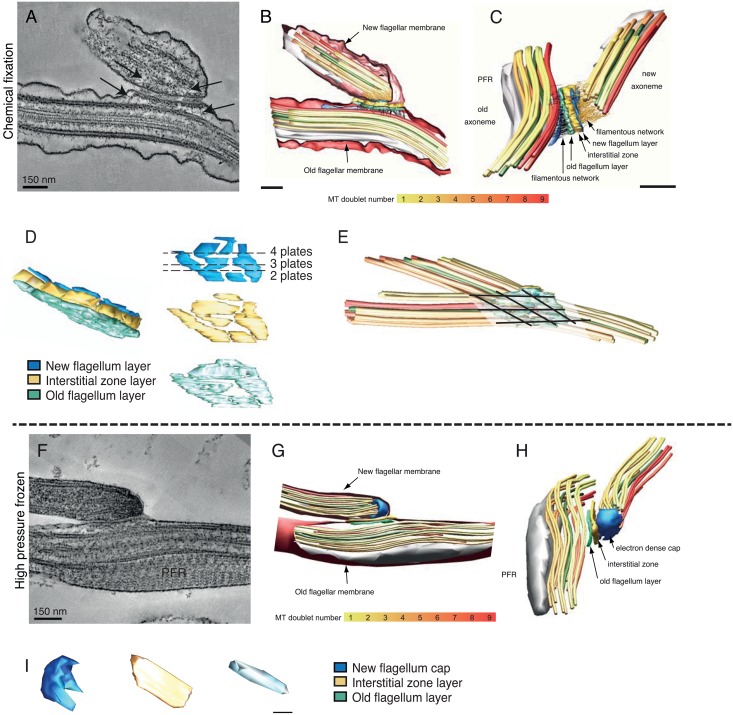
The 3D architecture of the FC region as showed by electron tomography. **A)** Tomographic slice of a chemically fixed sample showing the tri-laminar structure, its partition into plates and filamentous connectors to the two axonemes (arrows). **B)** 3D model of the two flagella in the FC region. The FC tri-laminar layer is shown in green (old flagellum layer), gold (interstitial zone layer), turquoise (new flagellum layer). The flagellar membranes are shown in pink, the central pair in green and the doublet microtubules are colour-coded according to the gradient shown below the models. **C)** 3D models of the axoneme near the flagella connector. The filamentous network is shown in gold and black. **D)** A 3D model of the tri-laminar structure first illustrated as a whole, and then separated into the individual layers. Numbers by the dotted lines show the number of plates that would be seen in a FC cross-section at this point. **E)** The orientation of the plates in the old and new flagellum FC layers are indicated by black lines. These lines appear parallel to the MTs of the underlying axoneme. **F**) A tomographic slice of a high pressure frozen flagella connector. The old flagellum and interstitial zone layers appear as continuously electron dense, instead of being partitioned into plates. In the new flagellum no layer is discernable, instead there is an electron dense cap. G) 3D model of the FC region (same color codes as above). Note the close proximity of the axoneme to the smooth flagellar membranes. H) In high pressure frozen samples no filamentous network is observable between the tri-laminar structure and the axonemes. I) Illustration of the three layers of the FC in a high pressure frozen cell.

A tomographic reconstruction of chemically fixed FCs was performed. A thin slice of one reconstruction (Fig 2A; S1 Movie) shows the tri-laminate structure and filamentous connections from it to both axonemes (arrows). A 3D model of the FC was produced by drawing around the structural features of interest in the tomogram. The top view (Fig 2B; S2 Movie) displays the flagellar membranes and shows how the tip of the new flagellum is apposed to the old flagellum. In the en face view (Fig 2C), membranous components were subtracted to visualise the microtubule doublets and the 3D morphology of the tri-laminar complex and its associated filamentous network (doublet microtubules are colour coded with a gradient from doublet 1 in pale yellow to doublet 9 in dark red).

Previously, each layer within the tri-laminar structure has been described as partitioned into three electron dense plates [14–18, 21]. However, in the tomographic reconstruction, the 3D structure of complete layers shows that they are subdivided into a range of 2–4 plates, of which 3 plates is the most common (Fig 2D). The 3D reconstruction also shows a correlation between the angles of partitions and the two axes underlying axonemes (Fig 2E).

The FC tri-laminar structure was less noticeable in the high pressure frozen material (Fig 2F; S3 Movie). A new flagellum plate was not detectable, probably because of the electron dense cap coating the entire inside of the new flagellum tip (Fig 2G–2H; S4 Movie). The interstitial layer was thinner than previously seen in the chemically fixed sample. Also here, the plates within the tri-laminar structure were not detectable (Fig 2I), neither was the filamentous network between the FC and the axonemes. Thus, the high pressure frozen FC reveals a more compact FC morphology with an electron dense cap instead of the new flagellum plate.

### The FC changes over the cell cycle

The time of progression of a cell through the cell cycle is directly correlated to the length of the new flagellum. We therefore measured the length of the new flagellum in those high pressure frozen cells in which the FC had been reconstructed.

**Fig 3:**
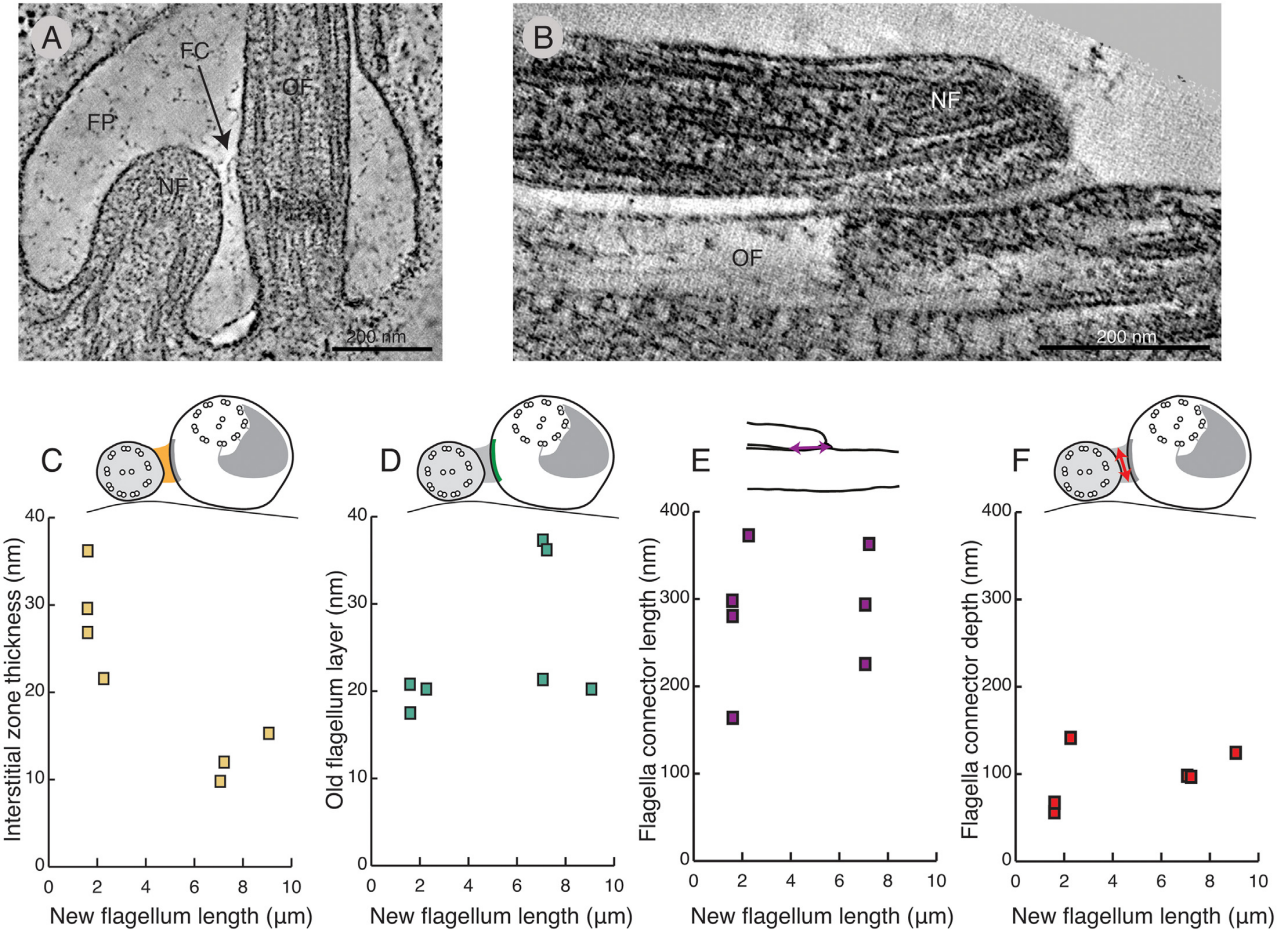
Structural maturation of the FC in high pressure frozen cells. **A)** A 3 nm thick tomographic slice of a forming FC on the side of an old flagellum. No physical connections to the 0.65 μm long new flagellum are observable at this stage. **B)** A 3 nm tomographic slice of a mature FC (7.7 μm long new flagellum). Note the thick electron dense layer inside the old flagellum. **C)** The interstitial zone thickness, as measured at its thinnest point, correlated with the new flagellar length. Note its thinning as the FC matures. **D-F)** The old flagellum FC layer thickness, the flagella connector length and depth seems variable but with no obvious correlation to cell cycle progression.

When the new flagella were short (below 2 μm), the FC was in the process of being formed inside the flagella pocket (Fig 3A; S5 Movie). At this stage, the FC was seen as a thin electron density in the interstitial zone extending from the wall of the old flagellum. We correlated the new flagellum length with the FC interstitial zone thickness (distance between the flagella) and found that this parameter decreased considerably as the FC matures (Fig 3C), being 28 ± 6 nm in short flagella (n = 4) and 13 ± 2 nm in longer flagella (Fig 3B; n = 4).

The structural maturation of the old flagellum FC layer during the cell cycle was, however, less clear (Fig 3D). The old flagellum layer in two cells with long new flagella had the same thickness (~20 nm) as found in cells earlier in the cell cycle; however two flagella connectors had old flagellum layers almost twice as thick (~35 nm). There was no difference in FC length (273 ± 61 nm; n = 9; Fig 3E) or depth (116 ± 30 nm; n = 8; Fig 3F) over the cell cycle.

We conclude that the thickness of the interstitial layer of the FC changes as the cell cycle progresses.

### The molecular arrangement of the interstitial zone layer

**Fig 4:**
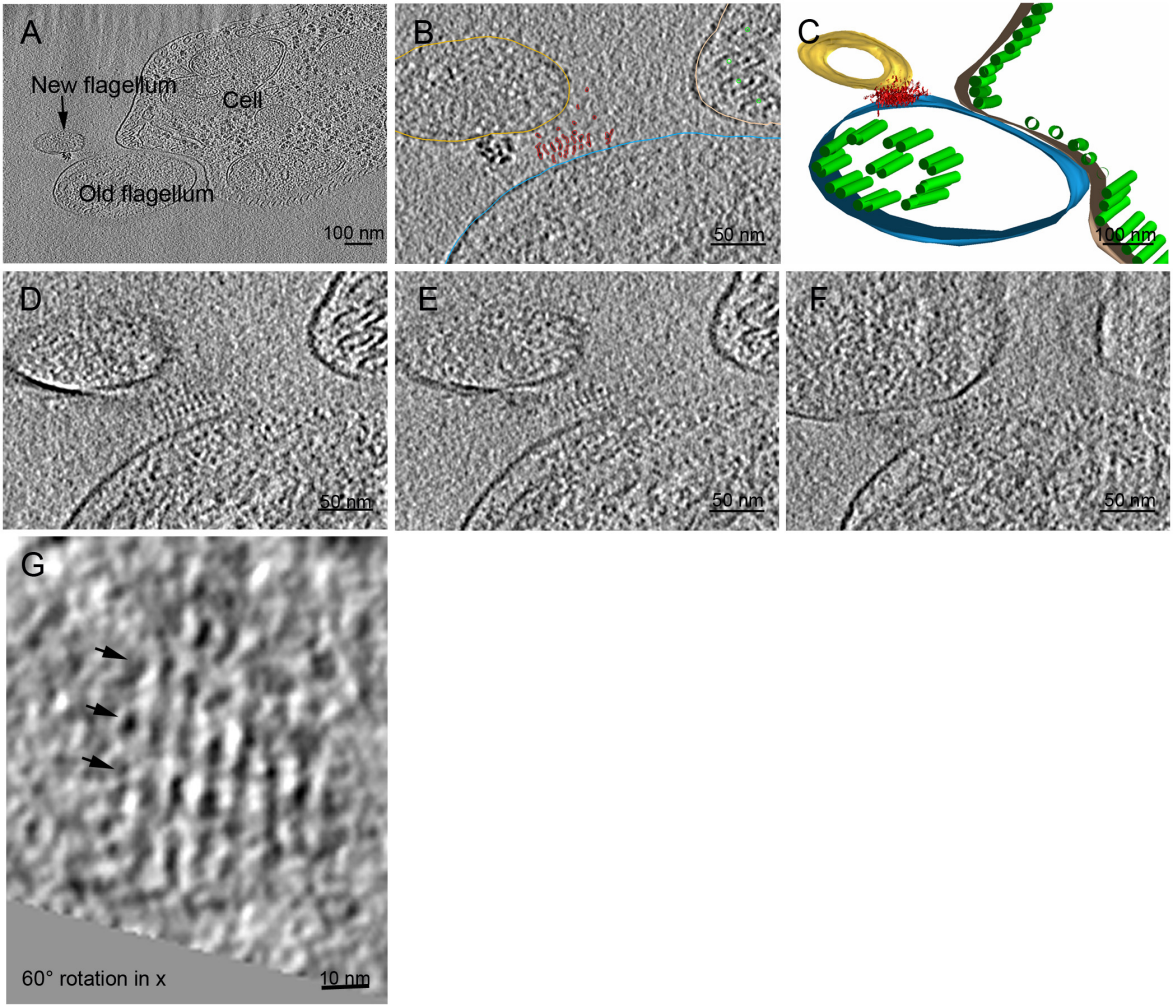
Cryo-electron tomography of a FC reveals linear, periodical protein arrangements in the interstitial zone. **A)** An overview of the cryo-section shows the old flagellum, new flagellum tip and the cell body sectioned transversally to the cells longitudinal axis. The electron dense particles close to the FC are of unknown nature and discussed further in S2 Fig. **B)** The objects of interest were traced on the tomographic slices: microtubules (green), the old flagellum (blue), new flagellum (gold), cell body (beige) and the densities found in the FC region (red) **C)** A 3D model of the tomogram reconstruction. Note the small diameter and cupped morphology of the new flagellum in comparison to the old flagellum, which indicates that this is the distal-most tip of the new flagellum. **D-F)** 15 nm thick slices from the tomogram reconstruction, note the increasing diameter of the new flagellum in E-F, as well as its decreasing distance to the old flagellum. This shows that the new flagellum distal tip is further from the old flagellum than the more proximal parts. In **D)** 3 layers of stacked electron dense periodical structures are found between the NF and OF. In **E)** this is reduced to two layers and in **F)** only one layer is seen. **G)** The periodical structures seen in E-F are parallel lines 7 nm apart when tilted 60° in x. These lines have periodically darker areas every 11 nm as indicated by the black arrowheads.

To image the FC protein architecture in a more native state, we made cryo-electron tomograms of vitreous sections. One electron tomogram of such a section, contained the most distal ~70 nm of the FC between the old and new flagellum, as well as the cell body (Fig 4A; S6 Movie). To examine the 3D architecture of the region we modelled the FC, membranes and microtubules (Fig 4B). The generated 3D model shows the complete FC structure, including membranes, as a ~100 nm wide connection between the old and new flagella (Fig 4C).

In views sliced through the tomogram, it is apparent that the distance between the old and new flagella is the greatest (~50 nm) closest to the distal tip of the new flagellum (Fig 4D); that this distance shrinks to ~20 nm as one moves more proximal in the new flagellum (Fig 4E–4F). The extracellular density we interpret as the FC lies close to the old flagellum throughout this volume, perhaps suggesting that it originates from that organelle. A very electron dense structure is also seen in close vicinity to the FC, which relevance we do not know, but a similar electron density was seen in a similar position in another cryo-electron microscopy image (Fig 4A–4B; S2 Fig). The FC layer in the new flagellum was not distinguishable, nor the axonemal microtubules in the new flagellum in this sample preparation. A faint density was visible in the old flagellum at the location of the FC. The dimensions measured in cryo-sectioned specimens have to be carefully interpreted due to compression, a characteristic artefact of vitreous sectioning [14,17,22]. The compression factor is estimated here at 50% based on the ovoid shape exhibited by the microtubules and the old flagellar membrane, and should be considered when reading the measurements here and in Table 1.

**Table 1:** Dimensions of the FC in different sample preparation methods.

	New flagellum layer (nm)	Interstitial zone layer (nm)	Old flagellum layer (nm)	FC width (nm)	Length (nm)	Depth (nm)
Negative stain [17]	16	18	13	90	400	N/A
Thin sections of chem. fix. [17]	20	32	15	N/A	N/A	N/A
Tomography of chem. fix. samples	21 (n = 2)	31 (n = 2)	29 (n = 2)	N/A	308 (n = 2)	187 (n = 2)
Tomography of high pressure frozen samples	N/A	20 (n = 10)	24 (n = 10)	N/A	272 (n = 9)	116 (n = 8)
Frozen hydrated section	N/A	20 (n = 1)	N/A	100 (n = 1)	N/A	N/A

The electron density that forms the interstitial FC component had a clear periodicity when seen in cross-section, even though there were tomographic slices where this periodicity was not as strong (possibly correlating to the areas indicated with blue boxes in Fig 1F’). When the distance between the flagella was larger (Fig 4D), three stacks of periodical electron densities were present. As the distance grew closer, this decreased to two (Fig 4E) and then one line (Fig 4F) of periodical densities.

When the tomogram was rotated, these periodical densities showed as parallel lines (Fig 4G) 7 nm apart, and with denser areas spaced 11 nm along them (arrows; Fig 4G). We conclude that cryo electron tomography of frozen hydrated sections of *T*. *brucei* cells has revealed the structural periodicity of the interstitial zone of the FC.

### The FC is consistently facing the same microtubule doublets on both sides of the junction

**Fig 5:**
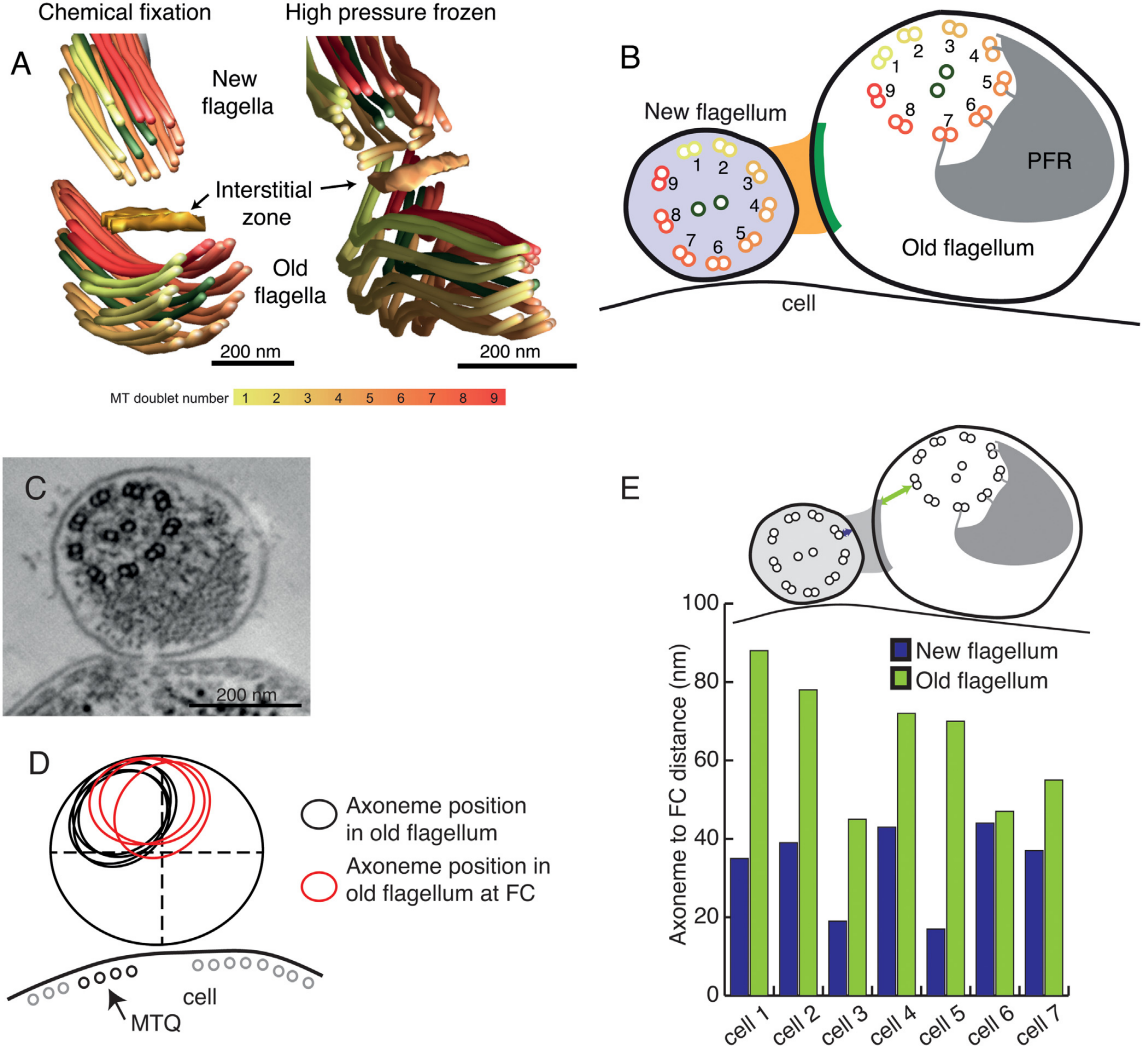
The FC displaces the old flagellum axoneme and its position is fixed in relation to both axonemes. **A)** 3D reconstructions of the FC region show it in close proximity to microtubule doublets 3–5 in the new flagellum and microtubule doublets 1, 7–9 in the old flagellum both in high pressure frozen and chemically fixed samples. **B)** A cartoon illustrating the FC and the axoneme orientations in cross-section. **C)** An example tomographic slice of a 30 nm thick flagellum cross-section, oriented with the microtubule quartet to the left, shows the axoneme located in the top-left corner of the flagellar space. **D)** The line drawing shows a few examples of axoneme positioning within the old flagellum (black ellipsoids), and at the flagella connector (red ellipsoids). **E)** The distances between the FC and the nearest doublet microtubule are longer in the old flagellum.

In the old *T*. *brucei* flagellum, defined numbers can be assigned to each doublet microtubule, as the central pair does not rotate [18,23] and a fixed, external structure, the paraflagellar rod (PFR), exists. The attachment of the axoneme to the PFR occurs at microtubule doublets 4–7 [19]. As previously described [14,17], the FC complex faces microtubule doublets number 7–9 in the old flagellum, but here we also see a close proximity between the FC and microtubule doublet 1. Previous studies were unable to distinguish which microtubule doublets the FC was facing in the new flagellum, or indeed if this side of the junction is more flexible, interacting with a range of microtubule doublets. Despite the apparent disorganisation inside the growing new flagellum tip[15], we have seen that the FC was consistently aligned with doublets 3–5 in the new flagellum (Fig 5A; n = 3). Thus, the FC follows specific microtubule doublets within both the new and old flagellum (Fig 5B).

### The FC progression shifts the axoneme to a more central location in the flagellum

To examine if the location of the axoneme in the old flagellum was perturbed by the passage of the FC, several images of flagellar cross-sections from multiple cells were aligned with the microtubule quartet in the sub-pellicular MT array to the bottom left of the flagellum (e.g. Fig 5C). An ellipsoid was placed where the axoneme was found in each image, which was invariably in the top left corner of the flagellum (Fig 5D). However, close to the FC, the position of the axoneme shifted ~100 nm away to a more central location (red ellipsoids). This shift in axonemal position around the FC can also be seen in longitudinal sections of the region (e.g. Fig 2F), and tells us that the FC presence rearranges the internal space of the old flagellum.

For several reasons, discussed later, it is possible that the filamentous network seen in chemically fixed cells between the FC and the axonemes represents a physical link between these three structures. We measured the distances the filamentous network would span, from the membrane of the flagellum to the closest doublet microtubule at the level of the FC. The membrane-axoneme distance was 61 ± 1 nm and 55 ± 9 nm (n = 2) respectively in the new and old flagella in chemically fixed cells. In high pressure frozen cells, the axoneme was found closer to the membrane in new flagella (33 ± 11 nm; n = 7), than in old flagella (65 ± 16 nm; n = 7; Fig 5C). Surprisingly, we also found a fibrous structure in this space between the FC and the axoneme in the old flagellum.

### The FC fibre, a novel component of the FC

**Fig 6:**
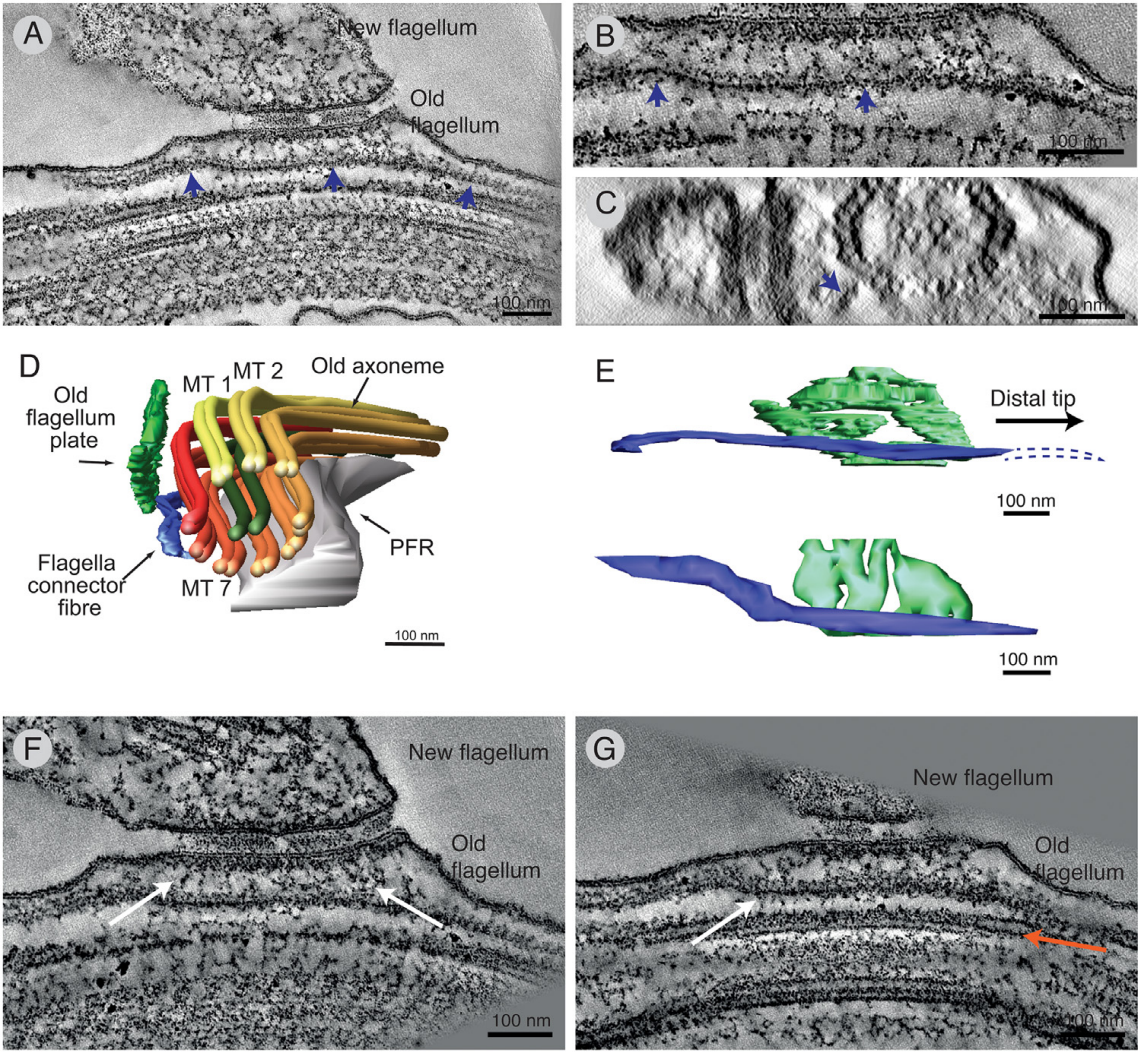
Chemical fixation reveals a novel filament inside the old flagellum at the FC. **A)** A 10 nm thick tomographic slice showing a long filamentous structure (blue arrows), the FC fibre, positioned close to the flagellar membrane, the old flagellum plate and the axoneme of the old flagellum. **B)** A 5 nm thick slice of the fibre reveals its non-hollow nature C) When rotated so as to display the axoneme in cross-section, it shows the FC fibre (blue arrow) located off-center from the FC and not within the closest distance between the FC and the axoneme. **D)** A 3D model of the old flagellum’s spatial relationship to the FC fibre shows its location between microtubule doublets 7–8. **E)** The FC fibre was ~900 nm long and extends beyond the FC in both the flagellum distal and proximal direction. The dotted line indicates that the FC left the reconstructed volume and therefore extended further than shown in the 3D model of the region. F) A tomographic slice showing the connections (white arrows) between the FC fibre and the old flagellum FC layer. G) Only a few connections (white arrow) were found between the FC fibre and the doublet microtubules (orange arrow).

In both chemically fixed electron tomograms of the FC, a novel, electron dense fibre was observed inside the old flagellum near the old FC layer and the flagellar membrane (Fig 6A). This fibre appears as a filament, ~20 nm by ~45 nm in cross-section and longer than the field of view in a single tomogram (Fig 6B). We have named this component the FC fibre. When tilted to show the flagellum in cross-section, the position of the FC fibre is off-centre towards the cellular side of the axoneme (Fig 6C). This is also shown in the 3D model, where the FC is consistently found between MT doublets 7 and 8 (Fig 6D). In tomograms of both FCs, the proximal extremity of the FC fibre initiates ~400 nm prior to the FC (Fig 6E). The complete length of the FC fibre (~870 nm) could only be measured in one tomogram, and its distal end extends ~200 nm further than the distal extremity of the old flagellum plate. The FC fibre had connections to the old flagellum FC plate (Fig 6F), but only a few connections to the doublet microtubules (Fig 6G).

## Discussion

**Fig 7:**
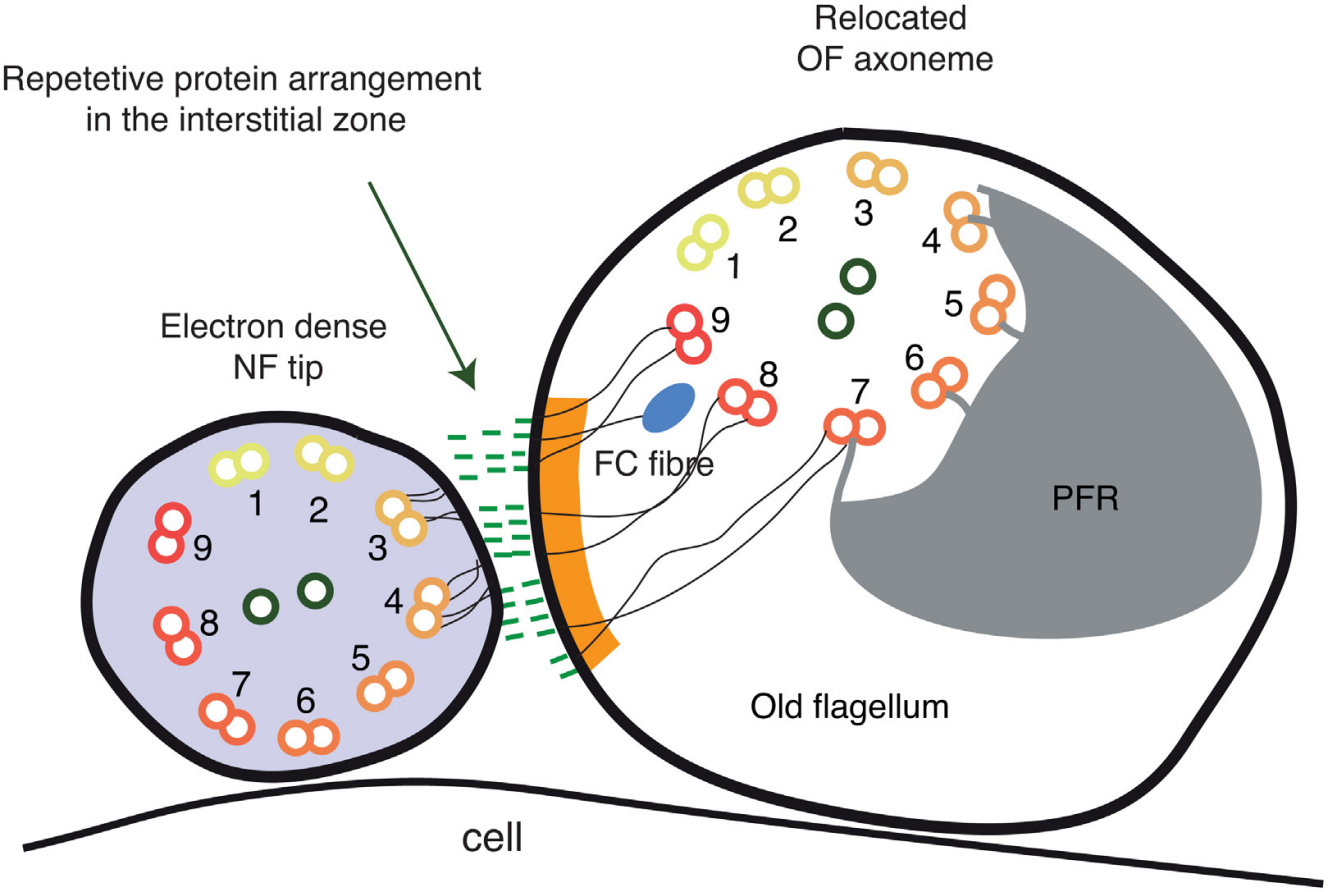
A schematic of the FC, combining published data and new data presented here.

In this paper, we have used a combination of fixation and imaging procedures to reveal the three dimensional ultrastructure of the FC of the procyclic form of *T*. *brucei*, a mobile cellular junction [14,17,18]. A combined detailed analysis of these data and previous publications on this structure shows that the FC behaves like a motorized double-sided vehicle that travels along microtubule doublets 7–9 in the old flagellum and in contact with microtubule doublets 3–5 in the new flagellum (Fig 7). Inside the new flagellum, the distance to the axoneme is 33 nm, a distance a kinesin molecule could easily span [24,25]. On the other side of the junction, the protein or protein complexes involved in linking the FC and the old axoneme must span the greater ~65 nm. This connection must also be very strong as it does not only move the FC, but also displaces the axoneme as it passes by.

We revealed structural variations of the FC and correlated them with the cell’s cycle stage judged by the length of the new flagellum. When the new flagellum is at, or close to, its stop point (at which the new flagellum tip stops translocating along the old flagellum[18]), the interstitial layer of the FC was reduced to about half the original thickness. The thickening of the old-flagellum layer of the FC in some of these flagella introduces the possibility of rearrangement of the external components. The maturation of the FC structure over the cell cycle is a novel finding and might hint towards the mechanism for removal of this structure after it has fulfilled its purpose.

A novel component of the FC was described—the FC-fibre. Because of this fibre’s morphology, length and location that all correlate well with those described of intraflagellar transport trains (IFT particles delivers flagellum building material to the flagellum tip using molecular motor proteins walking on the axonemal microtubules) [26–28], we suggest that it might represent a row of IFT particles (further discussion in S3 Fig and S1 Text).

This study also has methodological interest, as we studied the same structure with an unprecedented combination of electron microscopy methods. We showed that the high pressure frozen and freeze substituted FC appears more similar to the cryo-sectioned and cryo-visualised FC, with smooth flagellar membranes and unfragmented extracellular material. The thicknesses of the various FC plates vary depending on sample preparation (Table 1). Even though frozen hydrated sections of cells revealed the periodic organisation of the FC at the molecular level, cutting artefacts such as compression [22] added to the difficulty of visualizing the old and new flagellar plates especially when the FC plates are oriented perpendicular to the cutting direction. Therefore, all fixation methods are valuable for specific purposes, and quantitative results achieved using only one method should be interpreted with caution.

The FC structure provided by the 3D architecture presented here establishes a new level of insight into a junctional apparatus that possesses the capacity for lateral mobility. This insight into the FC substructure and morphogenesis is a necessary platform for future studies of molecular components and their assignment within the highly organized structure. In addition the structural definition will be critical for studies designed to reveal where the molecular motor is located and how it operates.

## Materials and Methods

### Sample preparation for room-temperature electron microscopy and tomography

Logarithmically growing procyclic 427 cells in SDM-79 medium were fixed by a) adding 2.5% glutaraldehyde to the culture or b) high pressure frozen using Leica EM Pact II (Leica Microsystems, Vienna) as in [15,29].

In brief, chemically fixed cells were postfixed (2.5% glutaraldehyde, 2% formaldehyde in 100 mM phosphate buffer pH 7–7.4 for 2 hours; then 1% osmium tetroxide in 100 mM phosphate buffer for 1–2 hours), en-bloc stained (2% magnesium uranyl acetate in water for 2 h) and dehydrated with increasing concentrations of ethanol, immersed in propylene oxide and infiltrated by increasing concentrations of epon.

High pressure frozen samples were freeze substituted (2% uranyl acetate from a 20% methanolic stock solution, in dehydrated acetone for 1 h). Infiltration with increasing concentration of HM20 (3:1, 2:1, 1:1, 1:3, 0:1 acetone:HM20 for several hours each) was performed at -50°C, where polymerization using UV light was initiated. Polymerization was finished with 48 h UV illumination at room temperature.

Thin sections (75 nm) were cut using an UltraCut microtome (Leica Microsystems,Vienna), and post stained with 3 min lead citrate only (chemically fixed samples), or 8 minutes 2% uranyl acetate followed by 3 minutes Reynold’s lead citrate (high pressure frozen samples) [30].

### Tomography

Sections 250–300 nm thick was cut, post-section stained and 15 nm colloidal gold particles (BBInternational, Cardiff, UK) was applied to both surfaces of the grid. Serial sections incorporating the entire FC were imaged using a Ultrascan 785 4k x 4k camera binned to 2k x 2k (Gatan, Pleasanton, CA, USA) every degree, ±60°, at 23000 x magnification on a F30 Tecnai microscope (FEI Company, Eindhoven, The Netherlands), then rotated 90° and a second axis was acquired. Pixel size was ~1 nm. Tomograms were reconstructed using the IMOD software [31], and 3D models were made by outlining objects of interest in the tomograms.

The lengths of new flagella were measured by taking lower magnification images of the thick serial sections containing the cell in which the FC had been imaged. Serial sections were aligned and 3D models of the new flagellum were made.

### Cryo-sectioning, cryo-electron microscopy and cryo-electron tomography

Cells were prepared by harvesting with centrifugation and resuspended in 20% dextran and 0.2% sucrose in medium. Within 3–4 minutes of resuspension, cells were high pressure frozen and then treated as in [12]. In brief, 80–100 nm thick frozen hydrated sections were cut and for tomography imaged every 1.5° and tilted ±60°on F20 Tecnai microscope (pixel size 0.76 nm; FEI Company, Eindhoven, The Netherlands). Fourier transform image was made from a subarea of a single 0.76 nm slice in IMOD.

## Supporting Information

S1 Fig Thin serial sections of two high pressure frozen flagella connectors. (TIF)

S2 Fig Extremely electron dense particles found in frozen hydrated sections. A) A 3 nm thick slice from the cryo-electron tomogram seen in Fig 4. The arrow points to a very electron dense structure close to the FC. B) A 5nm thick slice from the same tomogram as in A, revealing another very electron dense structure between the old flagellum and the cell body. C) A very electron dense structure (arrow) inside the cell body. D-F) Three different FCs showing no such electron density (D-E) and one with a very similar electron density to in A (F). G) A cell with two flagella showing an electron dense structure close to its flagellum.
(TIF)

S3 Fig FC fibre is likely an IFT train possibly important for docking the FC to its location on the old flagellum. **A)** Slice of an electron tomogram (20 nm thick) showing the FC fibre (white arrow) and a similar structure in the new flagellum (black arrow). **B)** Another structure similar to the FC fibre found very close to the FC in the new flagellum. **C)** A thin section micrograph of a flagellum with an internal electron dense particle (IFT; see box and white arrowheads). **D)** Cross sections of flagella showing the particles in the chemically fixed sample and their absence in high pressure frozen samples. **E)** The frequency of intraflagellar particles occurring in chemically fixed versus high pressure frozen cells. **F)** The particles show a biased localization towards the inter-MT spaces between MT doublets 3–4 and 7–8. **G)** Early during flagellar duplication, inside the flagellar pocket, a structure similar to the FC fibre is observed in the old flagellum. At this stage the FC is still rotating around the old flagellum [1]. **H)** A 3D model of the old flagellum with the 800 nm long fibre lying opposite to the FC between microtubules 7 and 8. **I)** The 3D model of the entire short new axoneme and the old axoneme with the associated fibre shows the stage of early flagellar duplication in this cell.
(TIF)

S1 Text Supplementary Results and Discussion. (DOCX)

S1 Movie The flagella connector (same as shown in Fig 2A–2E) in two serial sections of a chemically fixed sample. Each frame shows a 1nm thick slice of the tomogram reconstruction. 15 f.p.s. Scale bar 200nm.
(MOV)

S2 Movie 3D model of two serial sections of a chemically fixed flagellum containing a FC. All cellular components are coloured as in Fig 2. 15 f.p.s. Scale bar 200nm.
(MOV)

S3 Movie The flagella connector in a high pressure frozen sample (same as in Fig 3B). Each frame shows a 1nm thick slice of the tomogram reconstruction. 15 f.p.s. Scale bar 200nm.
(MOV)

S4 Movie 3D model of a high pressure frozen flagellum (same as shown in Fig 2F-2I). All cellular components are coloured as in Fig 2. 15 f.p.s. Scale bar 200nm.
(MOV)

S5 Movie Slices from an electron tomogram showing the newly formed FC between the short new flagellum and the side of the old flagellum. 6 fps. Scale bar 100 nm.
(MOV)

S6 Movie Flagella connector in two serial frozen hydrated sections. The movie begins more proximal in the flagellum and moves out to the new flagellum tip that ends in the volume. This is to our knowledge the first time serial electron tomography of frozen hydrated sections is published. 15 f.p.s. Scale bar 200nm.
(MOV)
