# Supplementary figures and images for: 3D Architecture of the Trypanosoma brucei Flagella Connector, a Mobile Transmembrane Junction
Source: PLoS Negl Trop Dis. 2016 Jan 28;10(1):e0004312. doi: 10.1371/journal.pntd.0004312 (PMC4731218; doi:10.1371/journal.pntd.0004312)

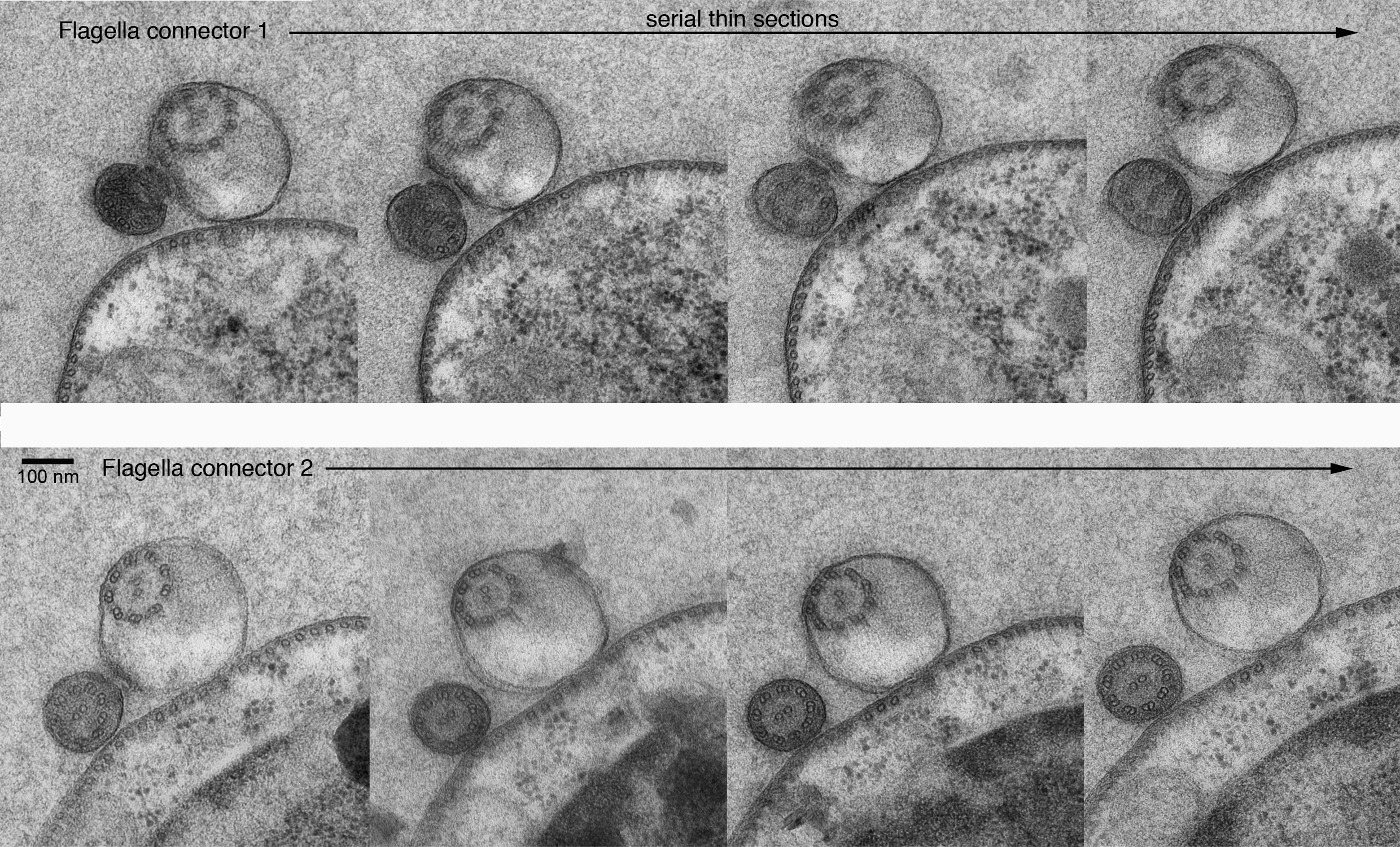

Supplement: S1 Fig — (TIF) [file pntd.0004312.s001.tif]

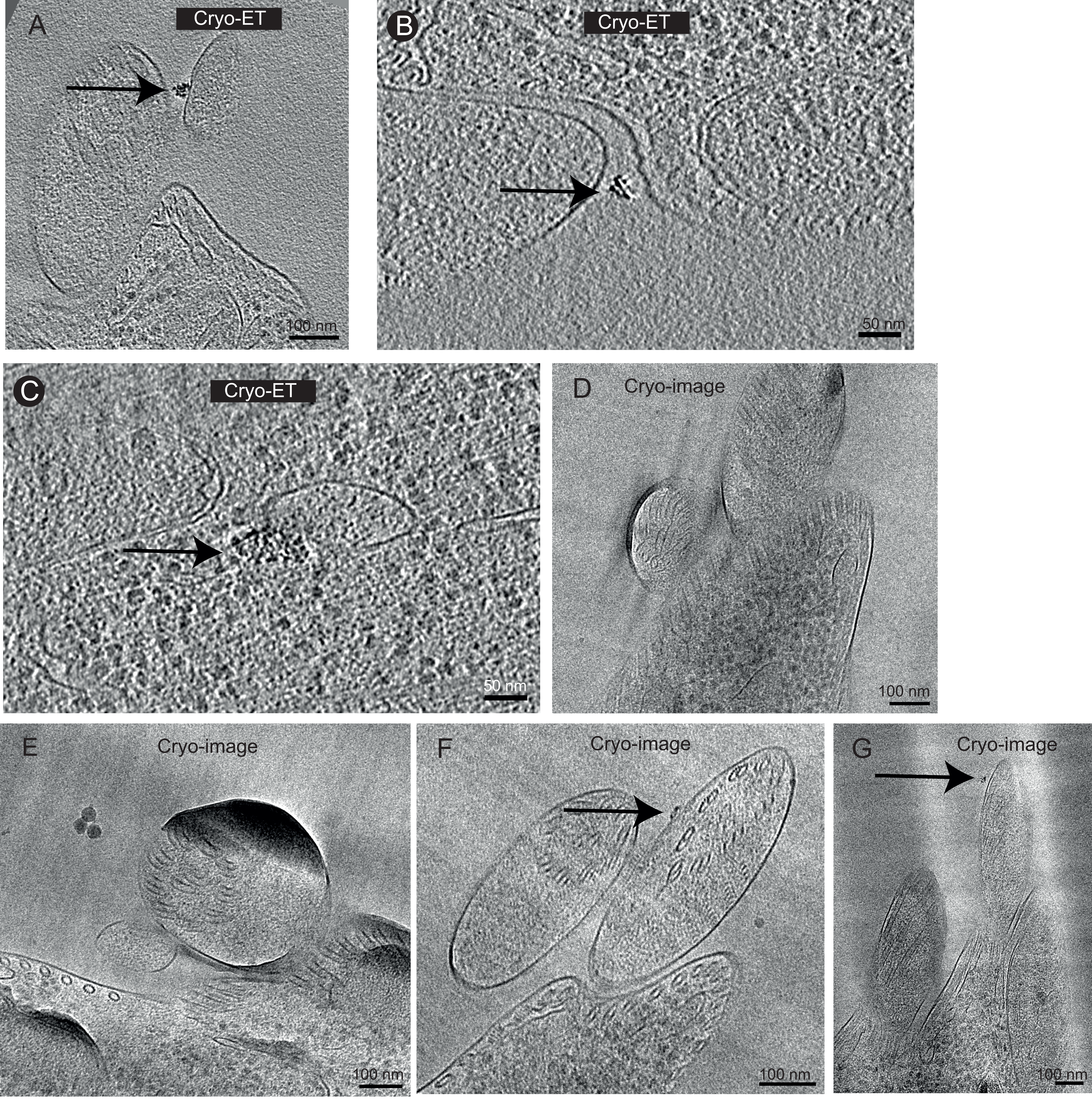

Supplement: S2 Fig — A) A 3 nm thick slice from the cryo-electron tomogram seen in Fig 4. The arrow points to a very electron dense structure close to the FC. B) A 5nm thick slice from the same tomogram as in A, revealing another very electron dense structure between the old flagellum and the cell body. C) A very electron dense structure (arrow) inside the cell body. D-F) Three different FCs showing no such electron density (D-E) and one with a very similar electron density to in A (F). G) A cell with two flagella showing an electron dense structure close to its flagellum. (TIF) [file pntd.0004312.s002.tif]

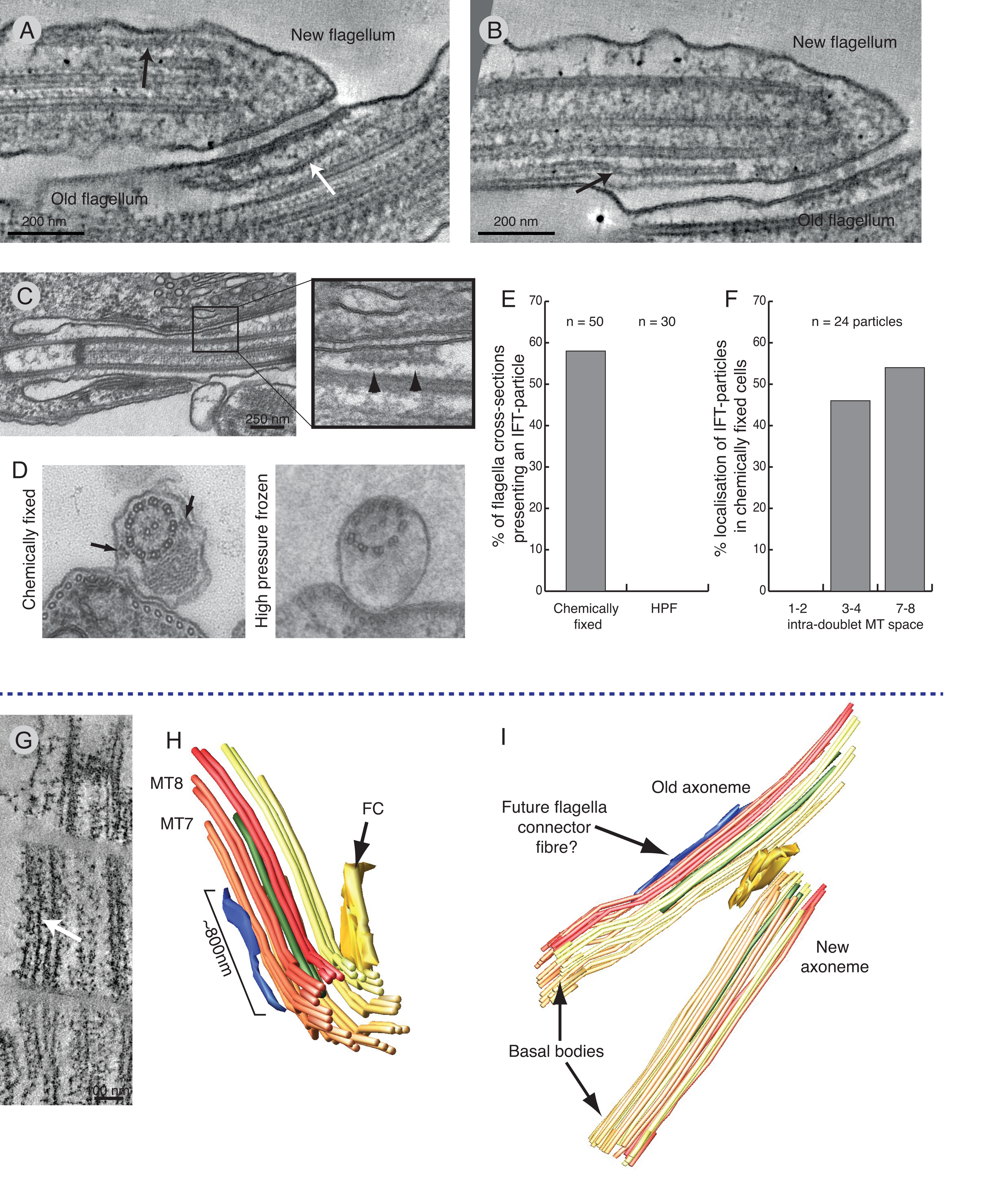

Supplement: S3 Fig — A) Slice of an electron tomogram (20 nm thick) showing the FC fibre (white arrow) and a similar structure in the new flagellum (black arrow). B) Another structure similar to the FC fibre found very close to the FC in the new flagellum. C) A thin section micrograph of a flagellum with an internal electron dense particle (IFT; see box and white arrowheads). D) Cross sections of flagella showing the particles in the chemically fixed sample and their absence in high pressure frozen samples. E) The frequency of intraflagellar particles occurring in chemically fixed versus high pressure frozen cells. F) The particles show a biased localization towards the inter-MT spaces between MT doublets 3–4 and 7–8. G) Early during flagellar duplication, inside the flagellar pocket, a structure similar to the FC fibre is observed in the old flagellum. At this stage the FC is still rotating around the old flagellum [1]. H) A 3D model of the old flagellum with the 800 nm long fibre lying opposite to the FC between microtubules 7 and 8. I) The 3D model of the entire short new axoneme and the old axoneme with the associated fibre shows the stage of early flagellar duplication in this cell. (TIF) [file pntd.0004312.s003.tif]
