# Supplementary material for: 3D Architecture of the Trypanosoma brucei Flagella Connector, a Mobile Transmembrane Junction
Source: PLoS Negl Trop Dis. 2016 Jan 28;10(1):e0004312. doi: 10.1371/journal.pntd.0004312 (PMC4731218; doi:10.1371/journal.pntd.0004312)
Supplement: S1 Text — (DOCX) [file pntd.0004312.s004.docx]

## Supplementary Results and Discussion

## Speculation into the nature and function of the FC fibre

Analysis of both electron micrographs and tomograms of chemically fixed cells revealed the presence of fibres of variable length attached to the plasma membrane, some of which were very similar to the FC fibre. We also found such fibres in the new flagellum, between doublets 3-4 and 8-9 (Figure S3A-B). These fibres also greatly resemble intraflagellar transport particles (IFT particles; Figure S3C; [2]). In thin flagellar cross sections, particles are seen in 60% of sections analysed (n=50 sections; Figure S3D-E). However, our analysis of over 30 cross sections of high pressure frozen flagella did not show similar structures (Figure S3D-E). This may be because the high pressure frozen flagellar lumen is commonly more dense than the chemically fixed samples, or because chemical fixation cross-links proteins more tightly, yielding complexes more electron dense. The (assumed) IFT particles found in the chemically fixed flagella were exclusively found opposite microtubule doublets 3-4 (54%) and 7-8 (46%; Figure S3F), in agreement with where IFT particles are found in *T. brucei* [3]. As the FC fibre also was found in between microtubule doublets 7-8, we suggest that the FC fibre may be an IFT particle train. Anterograde IFT in trypanosomes occurs at two different speeds 1.5 and 2.5 µm/s [5] whereas new flagella growth occurs at ~4µm/hour [6]. Therefore, it is possible that the FC fibre may be an IFT traffic jam caused by a fusion of slow-moving and fast moving IFT particles. This traffic jam could also consist of a modified IFT train that acts as an engine, pulling the new flagellum up along the old flagellum. However, as the FC transport is IFT independent [7], the FC fibre would then have to have additional motility to the normal anterograde kinesin II motility of IFT particles [8]. Because of the IFT independence of the FC motility [7], and the few physical links that we saw between the FC fibre and the axoneme, it seems unlikely that the FC fibre is an important component of FC transport.

The FC fibre may also have a more functional role in term of FC morphogenesis. The FC has initially two axes of motion that are later restricted during the cell cycle [1,4]. In a tomographic reconstruction of a dividing flagellar pocket, where the intracellular FC is still rotating around the old flagellum (opposite microtubule doublets 2-3-4; [1]), a similar structure to the FC fibre lies between microtubule doublets 7-8 (Figure S3G). We suggest that the FC fibre might physically prevent further rotation of the FC once it has reached microtubule doublets 7-8. However, to properly address this question, identification of the molecular components of the FC is required, and the motility of both the FC and IFT particles examined in their absence.

#### Supplementary references

1. Lacomble S, Vaughan S, Gadelha C, Morphew MK, Shaw MK, McIntosh JR, et al. Basal body movements orchestrate membrane organelle division and cell morphogenesis in Trypanosoma brucei. J Cell Sci. 2010;123: 2884–2891. doi:10.1242/jcs.074161

2. Pigino G, Geimer S, Lanzavecchia S, Paccagnini E, Cantele F, Diener DR, et al. Electron-tomographic analysis of intraflagellar transport particle trains in situ. J Cell Biol. 2009;187: 135–148. doi:10.1006/jsbi.1997.3934

3. Absalon S, Blisnick T, Kohl L, Toutirais G, Doré G, Julkowska D, et al. Intraflagellar transport and functional analysis of genes required for flagellum formation in trypanosomes. Mol Biol Cell. 2008;19: 929–944. doi:10.1091/mbc.E07-08-0749

4. Lacomble S, Vaughan S, Gadelha C, Morphew MK, Shaw MK, McIntosh JR, et al. Three-dimensional cellular architecture of the flagellar pocket and associated cytoskeleton in trypanosomes revealed by electron microscope tomography. J Cell Sci. 2009;122: 1081–1090. doi:10.1242/jcs.045740

5. Buisson J, Chenouard N, Lagache T, Blisnick T, Olivo-Marin JC, Bastin P. Intraflagellar transport proteins cycle between the flagellum and its base. J Cell Sci. 2013;126: 327–338. doi:10.1242/jcs.117069

6. Bastin P, MacRae TH, Francis SB, Matthews KR, Gull K. Flagellar morphogenesis: protein targeting and assembly in the paraflagellar rod of trypanosomes. Mol Cell Biol. 1999;19: 8191–8200.

7. Davidge J, Chambers E, Dickinson H, Towers K, Ginger ML, McKean PG, et al. Trypanosome IFT mutants provide insight into the motor location for mobility of the flagella connector and flagellar membrane formation. J Cell Sci. 2006;119: 3935–3943. doi:10.1242/jcs.03203

8. Kozminski KG, Beech PL, Rosenbaum JL. The Chlamydomonas kinesin-like protein FLA10 is involved in motility associated with the flagellar membrane. J Cell Biol. 1995;131: 1517–1527.
